# KIF26B Is Overexpressed in Medulloblastoma and Promotes Malignant Progression by Activating the PI3K/AKT Pathway

**DOI:** 10.1155/2022/2552397

**Published:** 2022-07-12

**Authors:** Yajun Liu, Xi Zhang, Ruihan Pan, Xiaolong Liang, Qichang Liu, Chao Yang, Xu Li

**Affiliations:** ^1^Department of Neurosurgery, The First Affiliated Hospital of Zhejiang University of Traditional Chinese Medicine, Hangzhou, Zhejiang, China; ^2^Department of Neurosurgery, The Second Affiliated Hospital of Zhejiang University School of Medicine, Hangzhou, Zhejiang, China

## Abstract

Medulloblastoma is one of the most common malignant tumors of the central nervous system in children. Although KIF2B was reported as an oncogene in several malignant tumor types, its role in medulloblastoma has not been studied so far. The PCR results of our study showed that KIF26B is highly expressed in medulloblastoma, and its high expression is associated with a high clinical stage. Knockdown the expression of KIF26B could significantly impair the proliferation and migration of medulloblastoma cells. KIF26B promotes the malignant progression of medulloblastoma by affecting the expression of phosphorylation of key proteins in the PI3K/AKT signaling pathway. With the help of 740 Y-P, activating the pi3k signaling pathway can partially rescue the phenotype. Therefore, our experimental results suggest that KIF26B is a potential target for medulloblastoma.

## 1. Introduction

Medulloblastoma (MB) is one of the most common central nervous system tumors in children and is extremely malignant [1]. Classified as grade 4 by the World Health Organization (WHO), it is the leading cause of death from childhood tumors. The incidence of MB accounts for approximately 16% of all childhood tumors [2]. Approximately 40% of all cerebellar tumors in childhood are MB, which tend to have a bimodal presentation between the ages of 3-9 and 8-9 years [3].

MB belongs to the primitive neuroectodermal tumors of the central nervous system, often originating from the cerebellar earthworm, and its clinical manifestations are mainly based on elevated intracranial pressure and cerebellar dysfunction, with a highly aggressive nature [4]. Medulloblastoma contains multiple molecular subtypes, and the most recent molecular typing studies currently classify medulloblastoma into four broad subtypes, namely, WNT, SHH, group 3, and group 4. Different molecular subtypes lead to different prognoses, and therefore, molecular typing has become an important basis for disease risk stratification [5, 6]. Unlike other intracranial tumors, MB can metastasize to the spinal cord through the cerebrospinal fluid or spread along the soft meninges, so the treatment effect is poor. Clinical treatment is based on neurosurgery with postoperative adjuvant radiotherapy and chemotherapy. However, patients are still more prone to recurrence after treatment and have a poor prognosis [7, 8]. Therefore, it is urgent to further understand the molecular mechanism of the development of MB and to find the target of treatment at the molecular level.

The human kinesin family member 26B (KIF26B) is a member of the kinesin family composed of 2108 amino acids. Recent studies have found that KIF26B gene contributes to tumorigenesis and endows certain cancers with malignant behavior. However, there is no relevant research on the expression and role of KIF26B in medulloblastoma. The human kinesin family member 26B (KIF26B) is a member of the kinesin family consisting of 2108 amino acids [9]. In the mouse model of embryogenesis, the KIF26B gene can participate in the development of limbs, face, and kidneys and play a very critical regulatory role [10]. Kinetic protein superfamily proteins (KIFs) are molecular motor proteins that bind to microtubules and are related to ATPase activity [11]. They are closely related to the mechanism of cancer development. Previous reports have shown that KIF1B in the KIF family is related to the occurrence and development of lung cancer and its prognosis and is related to central nervous system tumors and hepatocellular cancer metastasis [12]. The expression of KIF11 in the nucleus of prostate cells serves as a biomarker molecule for the treatment of prostate cancer [13]. Furthermore, recent studies have shown that high expression of KIF26B is associated with malignant expression and drug resistance in tumor cells. In colorectal cancer, the copy number of KIF26B gene was significantly associated with susceptibility to colorectal adenoma. KIF26B is highly expressed in colorectal cancer tissues, and the proliferative capacity of colorectal cancer cells was significantly reduced after KIF26B was silenced [10]. KIF26B can also act as an oncogene to activate the VEGF pathway and promote malignant progression of gastric cancer [14]. Knockdown of KIF26B was also found to inhibit the proliferation and invasion of breast cancer cells [15, 16]. However, the role of KIF26B in the development of MB is still unclear.

This study aims to determine the biological function and molecular mechanism of KIF26B in MB. The expression of KIF26B in MB tissues and cell lines was first detected. Afterwards, the role of KIF26B in MB cell proliferation, migration, and cell cycle was investigated. At last, the expression of PI3K/AKT pathway proteins was studied to explore the mechanism of KIF26B in medulloblastoma.

## 2. Materials and Methods

### 2.1. Patient Samples, Cell Lines, and Cell Culture

The clinical data and tumor samples of 30 MB patients who had received surgical resection from 2015 to 2020 at the First Affiliated Hospital of Zhejiang Chinese Medical University and the Second Affiliated Hospital of Zhejiang University School of Medicine, Hangzhou, Zhejiang Province, were collected. Histopathological classification was performed by two pathologists in a double-blind manner. All tumor specimens and adjacent normal brain specimens are frozen and preserved in liquid nitrogen until total RNA is extracted. Clinical features were collected in written form for each patient. Clinical data for the patients can be found in Table 1. All patients in this study obtained consent. The study was approved by the Ethics Committee of the First Affiliated Hospital of Zhejiang Chinese Medical University.

Human MB cell lines (D425 cells, D341 cells, D283 cells, and DAOY cells) were purchased from ATCC cell bank and the Cell Bank of the Chinese Academy of Sciences (Shanghai, China). All cells were cultured in RPMI1640 medium supplemented with 10% fetal bovine serum and penicillin/streptomycin. Cells were incubated at 37°C in the cell incubator with 5% CO_2_. When the confluence reached about 80%, the cells were subcultured. Only cells with good condition were used for experimental research.

### 2.2. siRNA Transfection

An appropriate amount of medulloblastoma cells was cultured in 6-well plates for 24 hours before transfection. Briefly, 2∗105 cells were seeded in 6-well plates, and siRNA at a final concentration of 100 nM was added to the medium and incubated at 37°C for 6 hours. Afterwards, the original medium was discarded, and fresh complete medium was added, and the culture was continued. RNA was collected 24 h after transfection, and protein was collected 48 h after transfection. Medulloblastoma cells were transfected with small interfering RNAs carrying KIF26B-specific sequences using Lipofectamine 2000 reagent (Invitrogen, USA) according to the manufacturer's instructions. Nonsense RNA interference served as a negative control (NC) for KIF26B siRNA. The transfection efficiency was detected by real-time quantitative RT-PCR and western blot. Two siRNAs of KIF26B were designed and bought from the Ribobio, Guangzhou, China, with the following sequences: siRNA-1 of KIF26B: 5′-CCACCUCUUUGAGAAGGATT-3′, siRNA-2 of KIF26B: 5′-CGGAC AGCCTCTCCTATTA-3′. The following nonsense siRNA was then used as a control: 5′- UUCUCCGAACGUGUCACGUTTTT-3′.

### 2.3. RNA Extraction and PCR

Total RNA was extracted from the obtained tissues and cultured cells with TRIzol reagent (Invitrogen, CA). RNA was reverse transcribed into cDNA with a reverse transcription kit (Takara) according to the manufacturer's instructions. SYBR Select Master Mix (Applied Biosystems, Cat: 4472908) was used for real-time PCR analysis. Primers for KIF26B and GAPDH are shown in Table 2. All PCR experiments were performed in a QuantStudioTM 6 Flex real-time PCR system. The reaction consisted of initial denaturation at 95°C for 10 min, 92°C for 15 s, and 60°C for 1 min, with 40 cycles. Finally, the relative expression levels of related mRNAs were calculated and normalized to GAPDH by the 2^-*Δ*Ct^ method.

### 2.4. Protein Extraction and Western Blot

MB cells were collected 48 h after transfection and proteins were extracted with RIPA lysis buffer (Kaiji, Nanjing, China) according to the protocol provided by the manufacturer. Equal amounts of proteins were then loaded onto SDS-page gels and transferred to PVDF membranes after electrophoresis. After being blocked with skim milk for 2 hours, the membranes were incubated with primary antibodies KIF26B (CAT#17422, 1 : 300, Proteintech, USA),GAPDH (CAT#5174, 1 : 1000, CST, USA), p-AKT (CAT#4060, 1 : 1000, CST, USA), mTOR (CAT#2983, 1 : 1000, CST, USA), and p-PI3K (CAT#4228, 1 : 1000, CST, USA) overnight. After washing 3 times with TBST, goat antirabbit HRP-conjugated secondary antibody (1 : 10,000; Abcam) or goat antimouse HRP-conjugated secondary antibody (1 : 10,000; left at room temperature for 2 h. ECL (Thermo Scientific) was used to detect the display bands, and all experiments were repeated 3 times.

### 2.5. Cell Proliferation Assay

The CCK8 detection kit (Beyotime Biotechnology, Shanghai, China) was used to detect cell proliferation according to the instructions. The procedure is as follows: First add 100 *μ*L medium with 2000 cells to each well of a 96-well plate. Then, add 10 *μ*L CCK8 reagent to each well, and place it in a CO_2_ incubator to incubate for 6 hours, and then shake to mix. The optical density (OD) value at 450 nm wavelength was then detected by the microplate reader. The experimental time gradients were 0, 24, 48, 72 h, 96 h, and 120 h, respectively. The experiment was repeated 3 times, and the average value was taken.

### 2.6. Colony Formation Assay

A number of 100 transfected cells were inoculated in fresh 6-well plates in medium containing 10% fetal bovine serum, and the medium was replaced every 3 or 4 days. After two weeks, cells were fixed in methanol and stained with 0.1% crystal violet. Visible colonies were counted manually.

### 2.7. Cell Migration Assay

Transwell chambers (Corning, USA) were used to detect cell migration. The chambers were placed in 24-well plates. Then, 100 *μ*L of serum-free medium was added to the upper chamber, first wetting the filter. 1 h later, 4,000 cells were added to 100 *μ*L of cell suspension, and 800 mL of 20% FBS 1640 medium was added to the lower chamber. The cells were then incubated in the incubator for 48 h. After 2 days, the cells were removed and fixed with 1 mL of 4% paraformaldehyde for 60 min at room temperature, followed by staining with 1 mL of 0.25% crystalline violet staining solution for 60 min.

### 2.8. Flow Cytometry Analysis

Transfected cells were cultured for 48 h and collected for flow cytometry analysis. Cells for cell cycle analysis were fixed in 75% alcohol, stored overnight at -20°C, and then subjected to FACScan analysis according to the cycling assay plus DNA kit (BD Biosciences) with oxypropane staining. The percentage of cells in G1, S, G2, and M phases was calculated and compared.

### 2.9. Statistics Analysis

Statistical analysis was performed with SPSS 20.0 and GraphPad Prism 9.0 software. The *t*-test was performed on samples from two groups, and one-way ANOVA was performed on samples from three or more groups to verify the differences between the experimental and control groups. *p* < 0.05 was considered that the differences were statistically significant.

## 3. Results

### 3.1. KIF26B Is Highly Expressed in Medulloblastoma Patient Tissues and Is Associated with Late Prognosis

**Figure 1:**
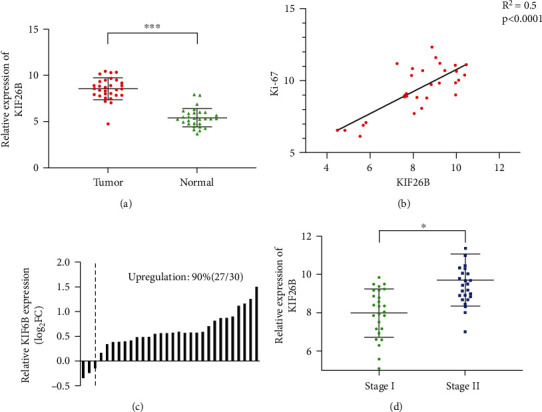
(a) The expression of KIF26B in the medulloblastoma patients. (b) Correlation analysis between the expression of KIF26B and KI67 in the medulloblastoma patients, *r*^2^ = 0.5, *p* < 0.01. (c) The relative expression of KIF26B in the patient's tumor specimens and para-tumor specimens. (d) The expression of KIF26B in patients with stage I medulloblastoma and patients with stage II medulloblastoma, *p* < 0.05.

The tissue samples were first used to analyze the expression of KIF26B in the tumor and adjacent normal tissue of 30 patients with medulloblastoma. As shown in Figure 1(a), the expression of KIF26B in medulloblastoma tumors is significantly up-regulated. We also found that the expression of KIF26B was closely related to KI67, a clinically recognized marker of tumor proliferation, indicating that KIF26B is closely related to tumor growth (Figure 1(b)). Then, we analyzed the expression ratio of KIF26B in each tumor sample and normal samples. As a result, we find that 27 of 30 patients had high expression of KIF26B, accounting for 90% of all patients, as shown in Figure 1(c).

In view of the high expression of KIF26B in medulloblastoma tumor tissues, we speculate that KIF26B may be involved in the malignant progression of medulloblastoma. Therefore, we analyzed the clinical data and analyzed the clinical phenotype within KIF26B expression. The results are shown in Figure 1(d). The expression of KIF26B in stage II patients was significantly higher than that in stage I.

These results suggest that the high expression of KIF26B is closely related to worse clinical manifestations and worse prognosis. Therefore, KIF26B is probably involved in the occurrence and development of medulloblastoma.

### 3.2. KIF26B Promotes the Proliferation and Migration of Medulloblastoma Cells

**Figure 2:**
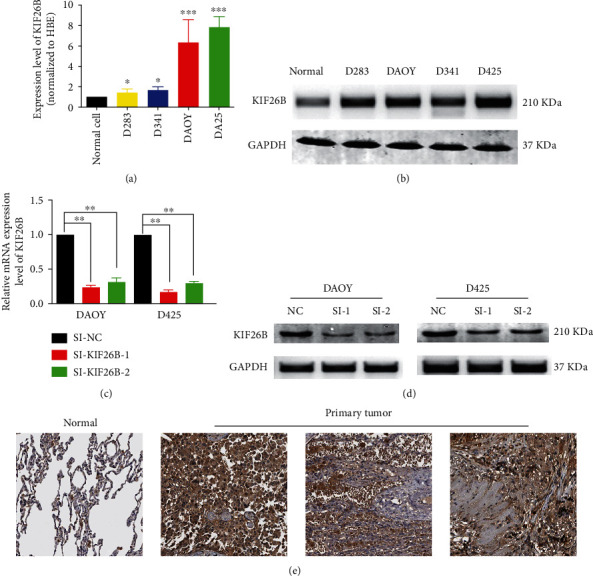
(a and b) PCR and western blot were used to detect the expression of KIF26B in the normal cell and medulloblastoma cell lines D283, D341, D425, and DAOY. (c and d) PCR and western blot were used to detect the expression of KIF26B in D425 and DAOY cell lines after being silenced by siRNA.

In order to select a suitable cell line for the experiment, we first determined the expression of KIF26B in several medulloblastoma cell lines relative to the normal cell. As the results shown in Figure 2(a), the expression of KIF26B in all the four cancer cell lines is significantly higher than that of normal cells. The western blot results also showed that the expression of KIF26B in the D425 and DAOY cell lines was significantly increased (Figure 2(b)). These 2 cell lines were used for the next experimental analysis.

We designed two small interfering RNAs targeting KIF26B to knock down the expression of KIF26B. After 48 hours of transfection with the transfection reagent, PCR and western blot were used to observe the expression of KIF26B in the knockdown group and the control group. As shown in Figure 2(c) and Figure 2(d), both SI-KIF26B-1 and SI-KIF26B-2 had achieved satisfactory inhibiting effects of the expression of KIF26B.

**Figure 3:**
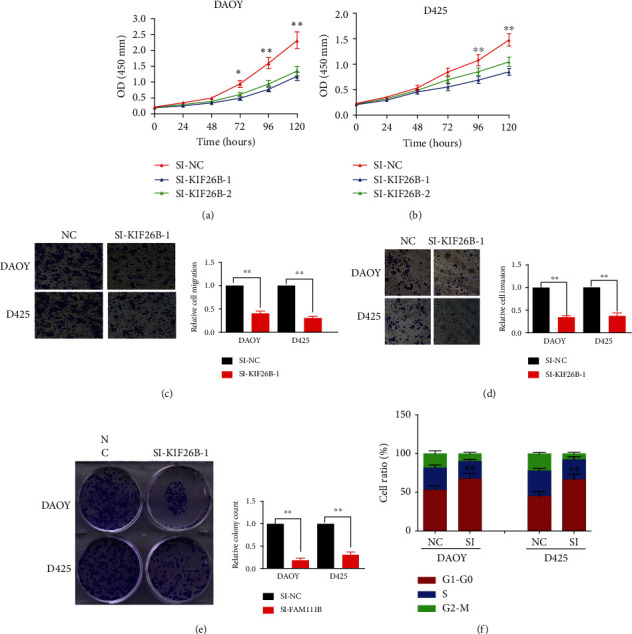
(a and b) The CCK8 experiment was used to observe the growth of D425 cell line and DAOY cell line after knocking down KIF26B. The absorbance value at 450 nm was observed and recorded every 24 h. (c and d) The transwell experiment was used to observe the migration of D425 cell line and DAOY cell line after knocking down KIF26B. The number of migrated cells were counted under a microscope and compared. (e) The clone formation experiment was used to observe the growth of D425 cells and DAOY cells after silencing KIF26B. The number of clones is recorded and compared. (f) After silencing KIF26B, the percentage of cells in G0-G1 phase, S phase, and M phase was observed by flow cytometry.

The malignant proliferation of tumor cells is one of the important reasons for the low cure rate. In order to further study the function of KIF26B, we explored whether knocking down endogenous KIF26B can inhibit the malignant proliferation of MB. The results of the CCK8 experiment showed (Figure 3(a) and Figure 3(b)) that after the expression of KIF26B was knocked down by siRNA, the proliferation ability of the D425 and DAOY cell lines had a significant downward trend compared with siNC after 24 hours. The results indicated that knockdown of endogenous KIF26B can inhibit the malignant proliferation of MB.

The transwell chamber was used to detect cell migration capabilities. The experimental results showed that compared with the control group, the migration and invasion ability of medulloblastoma cells in the KIF26B silenced group were significantly inhibited (Figures 3(c) and 3(d)), and the difference was statistically significant (*p* < 0.01).

In the clone formation experiment, as shown in Figure 3(e), we also find that the number of clones formed after the expression of KIF26B was knocked down and was significantly lower than that of the negative control group.

Finally, flow cytometry was used to analyze the cell cycle and detect the changes in the DNA ploidy content of each cell cycle in DAOY and D425 cells after knocking down the KIF26B gene expression. As shown in Figure 3(f), it can be seen from the experimental results that in the D425 and DAOY cell lines, the conversion from G0/G1 phase to S phase is inhibited. The cell cycle was blocked in G0/G1 phase.

### 3.3. KIF26B Promotes the Malignant Progression of Medulloblastoma through PI3K/AKT Pathway

**Figure 4:**
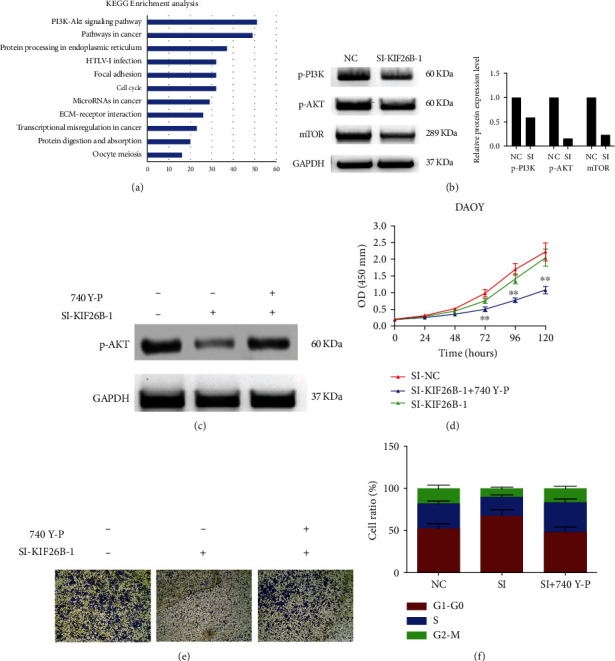
(a) Genes highly correlated with KIF26B expression were analyzed using KEGG enrichment analysis. The PI3K-Akt signal pathway ranks first. (b) Western blot was used to detect the expression of key proteins in the PI3K-AKT signaling pathway after knocking down KIF26B. (c) Western blot was used to detect the expression of phosphorylated AKT after knocking down KIF26B and adding PI3K pathway activator 740 Y-P with concentration of 20 *μ*M. (d, e, and f) After adding the PI3K pathway activator 740 Y-P (20 *μ*M), the proliferation (d), migration (e), and cell cycle (F) of DAOY cells were observed again.

In order to further explore the possible mechanism of KIF26B promoting the malignant progression of medulloblastoma, we first performed an enrichment analysis of the gene set that is significantly related to the expression of KIF26B. Briefly, genes with a KIF26B correlation greater than 0.4 were analyzed and collected and then submitted them to the DAVID bioinformatics analysis website for KEGG pathway analysis. The results are shown in Figure 4(a). As a result, most of the genes were enriched in the PI3K/AKT pathway, suggesting that KIF26B may exert its carcinogenic function by activating the PI3K/AKT pathway.

Therefore, we used western blot to observe the expression of key proteins in the PI3K/AKT pathway. The results are shown in Figure 4(b). After the expression of KIF26B was silenced, the expression of phosphorylated PI3K, AKT, and mTor was also significantly reduced.

In order to verify the influence of KIF26B on the PI3K pathway, we add the PI3K agonist 740 Y-P (20 nM) to the cells where KIF26B was inhibited, and the expression of phosphorylated AKT are higher as shown in Figure 4(c). The following functional experiments were performed again, and the result proved that after the expression of PI3K was reactivated, the proliferation and migration ability of DAOY cells were also rescued (Figure 4(d) and Figure 4(e)), while the cell cycle was also refrained from arrested from the G0/1 phase (Figure 4(f)).

## 4. Discussion

With the intensive study of cell biology and signaling pathways, it is widely accepted that genetic mutations and abnormal intracellular signaling are important for the development of medulloblastoma [17, 18]. Previous studies have shown that patients with medulloblastoma have a series of altered molecular genetic patterns such as deletion of the oncogene PTEN, p53 mutations, and abnormalities in SHH and WNT signaling pathways [19]. The prognosis of medulloblastoma patients with different molecular subtypes is very different, suggesting that our precise molecular subtyping has become an important basis for more effectively predicting the prognosis of medulloblastoma patients [6, 20].

The development and progression of medulloblastoma are a complex process involving abnormal transduction of intracellular signaling pathways and epigenetic changes [21]. Abnormal expression of core genes often leads to cell growth, differentiation, apoptosis, and migration disorders. Therefore, studying the core genes of abnormal expression is of great significance for the development of new targets for the treatment of medulloblastoma.

Based on these studies and the bioinformatics analysis, it is speculated that KIF26B is closely related to the malignant process of medulloblastoma. First, we detected the mRNA expression level of KIF26B in medulloblastoma tissues and adjacent normal tissues and found that KIF26B is highly overexpressed in tumor sample and patients with high KIF26B expression often have worse prognosis. This suggests that KIF26B may play an important role in the occurrence and development of MB. The following functional experiment verified our guess. After the expression of KIF26B was knocked down, the proliferation and migration capabilities of D425 and DAOY cells were significantly weakened. At the same time, the clone formation experiment also suggests that KIF26B can promote the clone formation ability of medulloblastoma cells. And the results of flow cytometry showed that KIF26B was retarded and the cell cycle was retarded in G0/1 phase. These experimental results showed that KIF26B can promote the malignant progression of medulloblastoma.

In order to further explore the mechanism of KIF26B promoting the malignant phenotype of medulloblastoma, we analyzed and collected gene sets that were significantly positively correlated with KIF26B expression and submitted them to the KEGG pathway for enrichment analysis. The results showed that most of the genes were enriched in the PI3K/AKT pathway.

Phosphatidyl/inositol 3-kinase/protein kinase B (PI3KAkt signaling pathway is closely related to cell survival, and it is found in most tumors that abnormal activation of this pathway plays an important role in tumorigenesis [22]. It is closely related to cell proliferation, apoptosis, and tumor angiogenesis. The pathway consists of three important protein molecules, namely, PI3K, Akt, and the downstream molecule mTOR. The role of PI3K changes in cancer development, progression, metastasis, and drug resistance. The PI3K-AKT signaling pathway, as an important pathway for regulating growth in cells, plays an important role in promoting the growth and survival of tumor cells after activation. After activation of Akt, it can achieve antiapoptotic effects by activating a variety of downstream molecules. PI3K Akt signaling pathway activation and tumor resistance are closely related, and the application of this pathway inhibitor, such as PI3K inhibitor, Akt inhibitor, and mTOR inhibitor, can reverse the resistance of tumor cells and restore the sensitivity of tumor cells to chemotherapeutic drugs. Different studies have proved the critical role of the entire PI3K/Akt pathway in the aggressiveness of several tumors, including cutaneous melanoma, breast cancer, and even medulloblastoma. In melanoma, the combination of PI3K and MAPK inhibitors can effectively reduce melanoma sensitivity of cells to treatment [23]. Gene mutations encoding PI3KCA can be used as independent prognostic predictors of breast cancer [24]. PI3K signaling pathway plays an important role in the activation of intracellular signaling pathways in embryonic cerebellum, and the inhibition of PI3K signaling pathway can significantly improve the inefficient medulloblastoma cell growth and promotion of stem cell-like MDB cell differentiation [25].

When we checked the literature, we also found that KIF26B can promote the progression of hepatocellular carcinoma by activating the PI3K/AKT pathway [26]. Therefore, we speculate that KIF26B may exert its carcinogenic function by activating the PI3K/AKT pathway. Then, we detected the expression of the key proteins in this pathway. And the results showed that after KIF26B was silenced, the expression of p-PI3K, p-AKT, and mTOR was also significantly reduced. After the PI3K activator 740 Y-P was used to reactivate the expression of PI3K, the phenotype suppressed by siRNA was rescued. This indicated that the effect of KIF26B on medulloblastoma cells is indeed carried out through the PI3K pathway.

Abnormal expression and inactivation of PTEN can promote the activation of PI3K/Akt signaling pathway, thereby further inhibiting the activity of GSK-3*β* and promoting the nuclear entry of *β*-catenin and the expression of its downstream target genes c-myc, Survivin, and CyclinD 1 [27]. As a cyclin protein, CyclinD1 can promote cell proliferation, and its expression has also been found to be increased in a variety of tumors including medulloblastoma [28]. Therefore, the proliferation of medulloblastoma cells can be inhibited by regulating the PTEN/PI3K/Akt signaling pathway. Our experimental results show that silencing of KIF26B can affect the cell cycle. At the same time, bioinformatics analysis found that the expression of KIF26B was also closely related to the expression of cell cycle-related proteins. This indicates that the abnormal expression of KIF26B may be related to the abnormal inactivation of PTEN.

## 5. Conclusion

In summary, our study shows that KIF26B is highly overexpressed in MB and is associated with worse clinical features and poor prognosis. KIF26B can promote the proliferation and migration of MB cells. In addition, KIF26B may participate in the malignant progression of medulloblastoma through PI3K/AKT signaling pathway.

## Figures and Tables

**Table 1:** Clinicopathological characteristics of patients with MB.

Clinicopathological	KIF26B expression		
Characteristics	Low	*High*	P value
Age			
≤3	9	7	
>3	7	7	0.73
Sex			
Female	6	8	
Male	10	6	0.28
WHO histological subtype			
Classic	12	11	
Desmoplastic	4	3	0.82
Tumor location			
Fourth ventricle	8	4	
Outside fourth ventricle	6	10	0.13
Residual tumor size			
≤1.5 cm^2^	13	8	
>1.5 cm^2^	3	6	0.15

**Table 2:** All primer sequence used in this research.

Gene	Sense	Anti-sense
KIF26B	CCACCUCUUUGAGAAGGATT	UUCCUUCUCAAAGAGGUGGTT
GAPDH	GACAGTCAGCCCGCATCTTCT	TTAAAAGCAGCCCTGGTGAC

## Data Availability

Data is available on request.

## References

[1] Liu X., Ding C., Tan W., Zhang A. (2020). Medulloblastoma: molecular understanding, treatment evolution, and new developments. *Pharmacology & Therapeutics*.

[2] Quinlan A., Rizzolo D. (2017). Understanding medulloblastoma. *JAAPA*.

[3] Ramaswamy V., Remke M., Bouffet E. (2016). Risk stratification of childhood medulloblastoma in the molecular era: the current consensus. *Acta Neuropathologica*.

[4] Gerber N. U., Mynarek M., von Hoff K., Friedrich C., Resch A., Rutkowski S. (2014). Recent developments and current concepts in medulloblastoma. *Cancer Treatment Reviews*.

[5] Juraschka K., Taylor M. D. (2019). Medulloblastoma in the age of molecular subgroups: a review. *Journal of Neurosurgery. Pediatrics*.

[6] Northcott P. A., Korshunov A., Witt H. (2011). Medulloblastoma comprises four distinct molecular variants. *Journal of Clinical Oncology*.

[7] Bleil C. B., Bizzi J. W. J., Bedin A., de Oliveira F. H., Antunes A. C. M. (2019). Survival and prognostic factors in childhood medulloblastoma: a Brazilian single center experience from 1995 to 2016. *Surgical Neurology International*.

[8] Cassia G. S. E., Alves C., Taranath A. (2018). Childhood medulloblastoma revisited. *Topics in Magnetic Resonance Imaging*.

[9] Uchiyama Y., Sakaguchi M., Terabayashi T. (2010). Kif26b, a kinesin family gene, regulates adhesion of the embryonic kidney mesenchyme. *Proceedings of the National Academy of Sciences of the United States of America*.

[10] Wang J., Cui F., Wang X. (2015). Elevated kinesin family member 26B is a prognostic biomarker and a potential therapeutic target for colorectal cancer. *Journal of Experimental & Clinical Cancer Research*.

[11] Aponte-Lopez A., Fuentes-Panana E. M., Cortes-Munoz D., Munoz-Cruz S. (2018). Mast cell, the neglected member of the tumor microenvironment: role in breast cancer. *Journal of Immunology Research*.

[12] Yang S. Z., Wang J. T., Yu W. W., Liu Q., Wu Y. F., Chen S. G. (2015). Downregulation of KIF1B mRNA in hepatocellular carcinoma tissues correlates with poor prognosis. *World Journal of Gastroenterology*.

[13] Wissing M. D., De Morree E. S., Dezentje V. O. (2014). Nuclear Eg5 (kinesin spindle protein) expression predicts docetaxel response and prostate cancer aggressiveness. *Oncotarget*.

[14] Zhang Z., Chen G. (2020). A logical relationship for schizophrenia, bipolar, and major depressive disorder. Part 1: evidence from chromosome 1 high density association screen. *Journal of Comparative Neurology*.

[15] Wang Q., Zhao Z. B., Wang G. (2013). High expression of KIF26B in breast cancer associates with poor prognosis. *PLoS One*.

[16] Gu S., Liang H., Qi D. (2018). Knockdown of KIF26B inhibits breast cancer cell proliferation, migration, and invasion. *Oncotargets and Therapy*.

[17] Pugh T. J., Weeraratne S. D., Archer T. C. (2012). Medulloblastoma exome sequencing uncovers subtype-specific somatic mutations. *Nature*.

[18] Cho Y. J., Tsherniak A., Tamayo P. (2011). Integrative genomic analysis of medulloblastoma identifies a molecular subgroup that drives poor clinical outcome. *Journal of Clinical Oncology*.

[19] Kagawa N., Maruno M., Suzuki T. (2006). Detection of genetic and chromosomal aberrations in medulloblastomas and primitive neuroectodermal tumors with DNA microarrays. *Brain Tumor Pathology*.

[20] Borowska A., Jozwiak J. (2016). Medulloblastoma: molecular pathways and histopathological classification. *Archives of Medical Science*.

[21] Blandin Knight S., Crosbie P. A., Balata H., Chudziak J., Hussell T., Dive C. (2017). Progress and prospects of early detection in lung cancer. *Open Biology*.

[22] Hoxhaj G., Manning B. D. (2020). The PI3K-AKT network at the interface of oncogenic signalling and cancer metabolism. *Nature Reviews. Cancer*.

[23] Candido S., Salemi R., Piccinin S., Falzone L., Libra M. (2022). The PIK3CA H1047R mutation confers resistance to BRAF and MEK inhibitors in A375 melanoma cells through the cross-activation of MAPK and PI3K-Akt pathways. *Pharmaceutics*.

[24] Sobhani N., Roviello G., Corona S. P. (2018). The prognostic value of PI3K mutational status in breast cancer: a meta- analysis. *Journal of Cellular Biochemistry*.

[25] Frasson C., Rampazzo E., Accordi B. (2015). Inhibition of PI3K signalling selectively affects medulloblastoma cancer stem cells. *BioMed Research International*.

[26] Li H., Shen S., Chen X., Ren Z., Li Z., Yu Z. (2019). miR-450b-5p loss mediated KIF26B activation promoted hepatocellular carcinoma progression by activating PI3K/AKT pathway. *Cancer Cell International*.

[27] Carracedo A., Pandolfi P. P. (2008). The PTEN-PI3K pathway: of feedbacks and cross-talks. *Oncogene*.

[28] Asuthkar S., Venkataraman S., Avilala J. (2022). SMYD3 promotes cell cycle progression by inducing cyclin D3 transcription and stabilizing the cyclin D1 protein in medulloblastoma. *Cancers*.

